# Metastatic Non‐Myofibroblastic Sarcoma Harbouring *EML4‐ALK* Fusion—Dramatic Response to ALK Tyrosine Kinase Inhibitors and Development of Resistance Mutations

**DOI:** 10.1002/cnr2.2164

**Published:** 2024-08-26

**Authors:** Isabella Wilson, Min Qiu, Malinda Itchins, Bin Wang, Min Li Huang, Peter Grimison

**Affiliations:** ^1^ Department of Medical Oncology Chris O'Brien Lifehouse Sydney Australia; ^2^ Faculty of Medicine and Health The University of Sydney Sydney Australia; ^3^ Sydpath, St Vincent's Hospital Sydney Australia; ^4^ St Vincent's Clinical School University of New South Wales Sydney Australia; ^5^ Department of Medical Oncology Royal North Shore Hospital Sydney Australia; ^6^ Kinghorn Centre for Cancer Genomics Medicine, Garvan Institute of Medical Research Sydney Australia

**Keywords:** alectinib, anaplastic lymphoma kinase, lorlatinib, sarcoma, tyrosine kinase inhibitors

## Abstract

**Background:**

Anaplastic lymphoma kinase (*ALK*) rearrangements are rare in non‐myofibroblastic sarcoma and there is limited data on the efficacy of ALK tyrosine kinase inhibitors (TKIs) and mechanisms of resistance in these patients.

**Case:**

A 58 year‐old man with metastatic non‐myofibroblastic sarcoma was found to have an *EML4‐ALK* fusion on molecular sequencing. After progression on first line systemic therapy with doxorubicin, the patient received alectinib, a second generation ALK inhibitor, and had a marked clinical and radiological response. He progressed after 5 months of treatment. Repeat lung biopsy identified the emergence of an *ALK* I1171N resistance mutation. He was then treated with lorlatinib, again with rapid clinical improvement and significant partial radiological response. He progressed after 4 months, at which time a repeat lung biopsy identified a new *ALK* kinase domain mutation G1202R. The patient was subsequently treated with chemotherapy, though unfortunately died shortly after due to rapidly progressive disease.

**Conclusion:**

This case report adds to a body of evidence demonstrating the potential transformative response to targeted therapy in non‐lung solid organ tumours harbouring *ALK* fusions. This is the first description tracking the development of resistance mutations in a patient with non‐myofibroblastic sarcoma and questions the utility of the presence of G1202R mutation as a marker of lorlatinib sensitivity in non‐lung *ALK* rearranged tumours, contrary to experience in lung cancer.

## 1 Introduction

Alterations in the anaplastic lymphoma kinase (*ALK*) gene including point mutations, deletions and fusions play an oncogenic role in several solid organ and haematological malignancies [1]. Fusion with the echinoderm microtubule‐associated protein‐like 4 (*EML4*) is the most common ALK fusion in non‐small cell lung cancer (NSCLC) [1]. *ALK* fusions have oncogenic potential because of constitutive activation of intracellular kinase pathways such as downstream RAS, PI3K and JAK3 [2]. *ALK* fusions are an established oncogenic target in the treatment of NSCLC, and there are numerous tyrosine kinase inhibitors (TKIs) currently approved for use. There is limited prospective data to support the use of ALK TKIs in other solid organ tumours. Here, we describe the case of a patient with metastatic non‐myofibroblastic sarcoma harbouring an *EML4‐ALK* fusion, encountering marked sequential response to alectinib and lorlatinib.

## 2 Case

A 58 year‐old man with no significant past medical history was diagnosed with an advanced metastatic non‐myofibroblastic sarcoma of soft tissue in 2021 at Chris O'Brien Lifehouse, Sydney, Australia. The presenting symptom was painless left shoulder swelling. An MRI demonstrated a 170 × 110 × 150 mm heterogeneous mass overlying and involving the scapula. Positron emission tomography (PET) scan identified bilateral lung metastases up to 13 mm in size. The patient underwent palliative left partial scapulectomy in July 2021.

**FIGURE 1:**
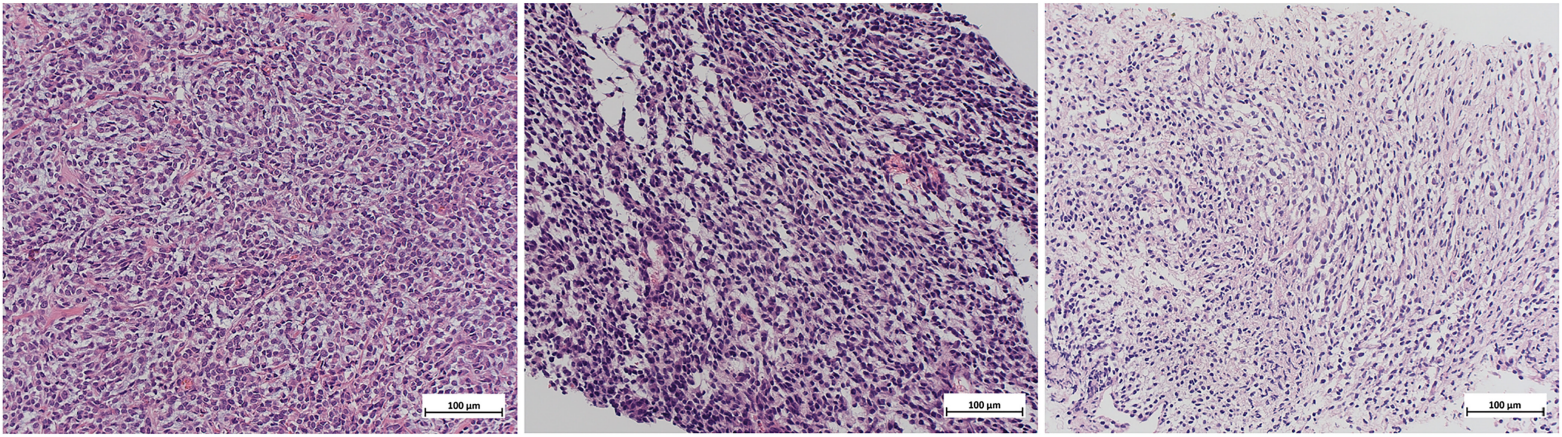
Morphology of the tumour at 20x magnification from the initial resection specimen (left), biopsy post alectinib (middle) and biopsy post lorlatinib (right).

Histopathology revealed a high grade sarcomatoid malignancy and immunohistochemistry for pan‐CK, CD99, desmin, myogenin, MyoD1, BRAF, NRAS and Melan A were negative (Figure 1). FISH was negative for SS18, CIC, DDIT3, NR4A3 and NRTK. FISH was positive for EWSR1; however, the cancer was not in keeping with an Ewing's sarcoma morphologically. The case was reviewed in a tertiary sarcoma multidisciplinary team meeting with a consensus diagnosis of non‐myofibroblastic sarcoma.

Molecular sequencing using the Illumina TruSight Oncology 500 panel revealed a fusion between *EML4* and *ALK* (V5, *EML4* exon 2—*ALK* exon 20), as well as high *NTRK1* RNA expression, gain of function mutation in oncogene TERT, biallelic loss of tumour suppressor *CDKN2A* and *CDKN2B*, microsatellite stable and low tumour mutation burden (0.8 mut/Mb).

Prior to receiving molecular sequencing results (available January 2022), the patient received standard first line palliative chemotherapy with doxorubicin for six cycles between August and December 2021. PET scan after two cycles showed stable disease. PET scan in January 2022 after six cycles showed marked progression of disease with increase in size and number of lung metastases measuring up to 67 mm, associated with progressive dyspnoea.

**FIGURE 2:**
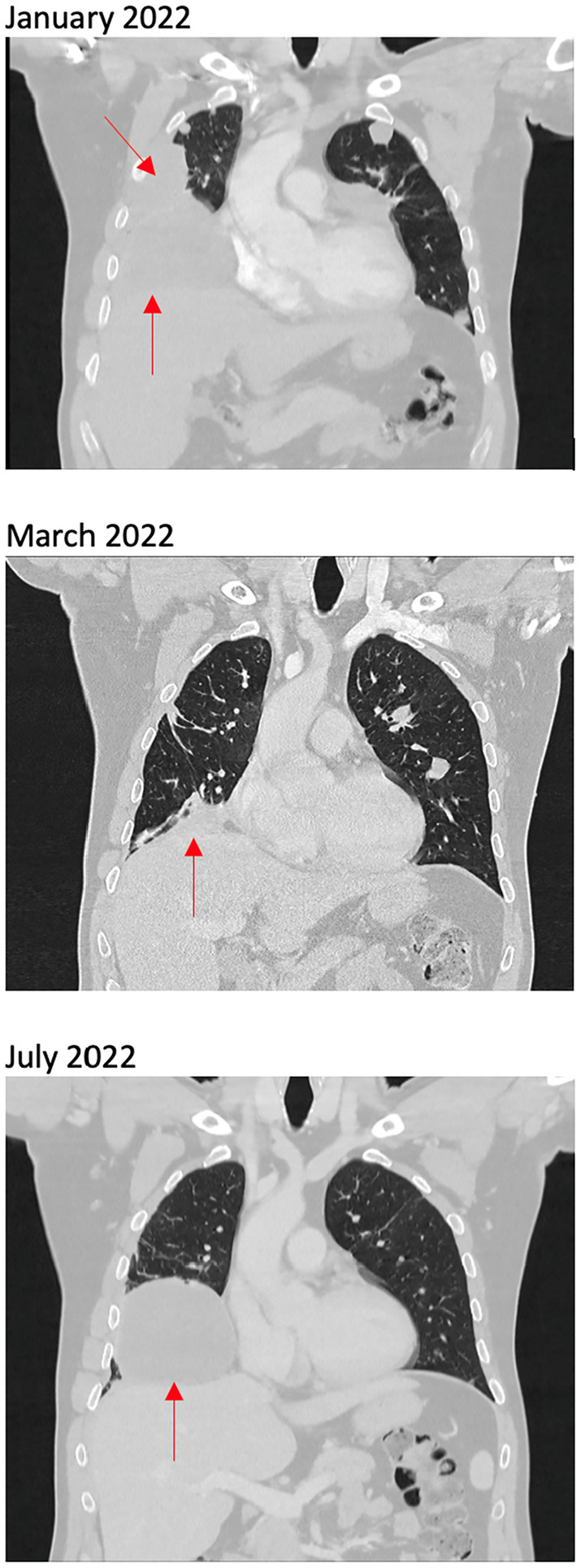
CT scan at baseline, during response to alectinib, and at progression.

The patient enrolled in a phase II clinical trial of alectinib for patients with solid organ tumours harbouring an *ALK* gene alteration (MoST 14 sub‐study 32, ACTRN12621000312842). He commenced alectinib in February 2022 and experienced a dramatic response to therapy with symptom improvement within 1 day of commencing treatment. CT scan in March 2022 after 6 weeks of treatment revealed partial response by RECIST criteria with dramatic decrease in size of lung metastases, with the largest lesion in the right middle lobe reducing from 103 to 28 mm (Figure 2). He remained on alectinib until July 2022, at which point he had rapid progression of lung metastases both clinically and radiologically (Figure 2). He required hospital admission for drainage of malignant pleural effusion and management of respiratory failure.

**FIGURE 3:**
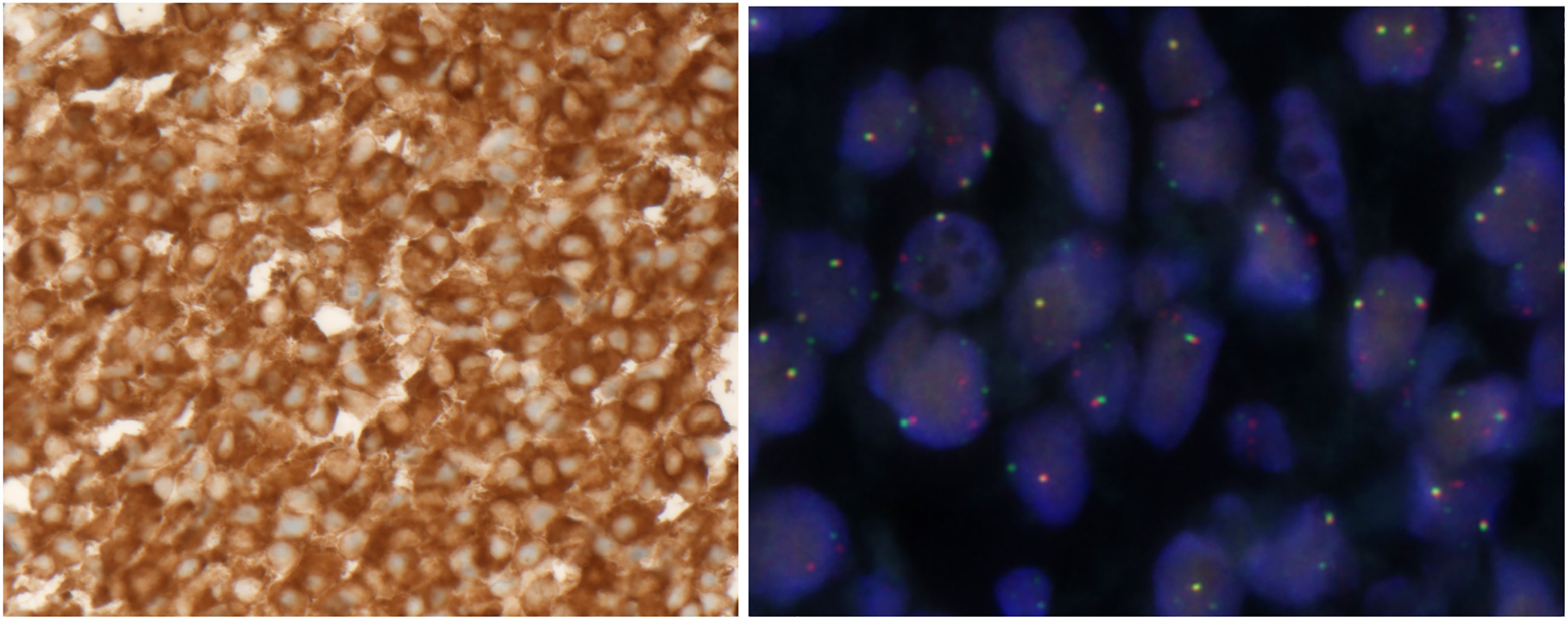
Positive ALK immunohistochemistry at 40x magnification (ALK D5F3 clone, Cell Signalling) (left). Interphase fluorescence in situ hybridization (FISH) confirming the presence of ALK gene rearrangement (Vysis LSI Dual Colour Break Apart Rearrangement Probe with cut off value of 15% used to determine FISH positivity) (right).

**FIGURE 4:**
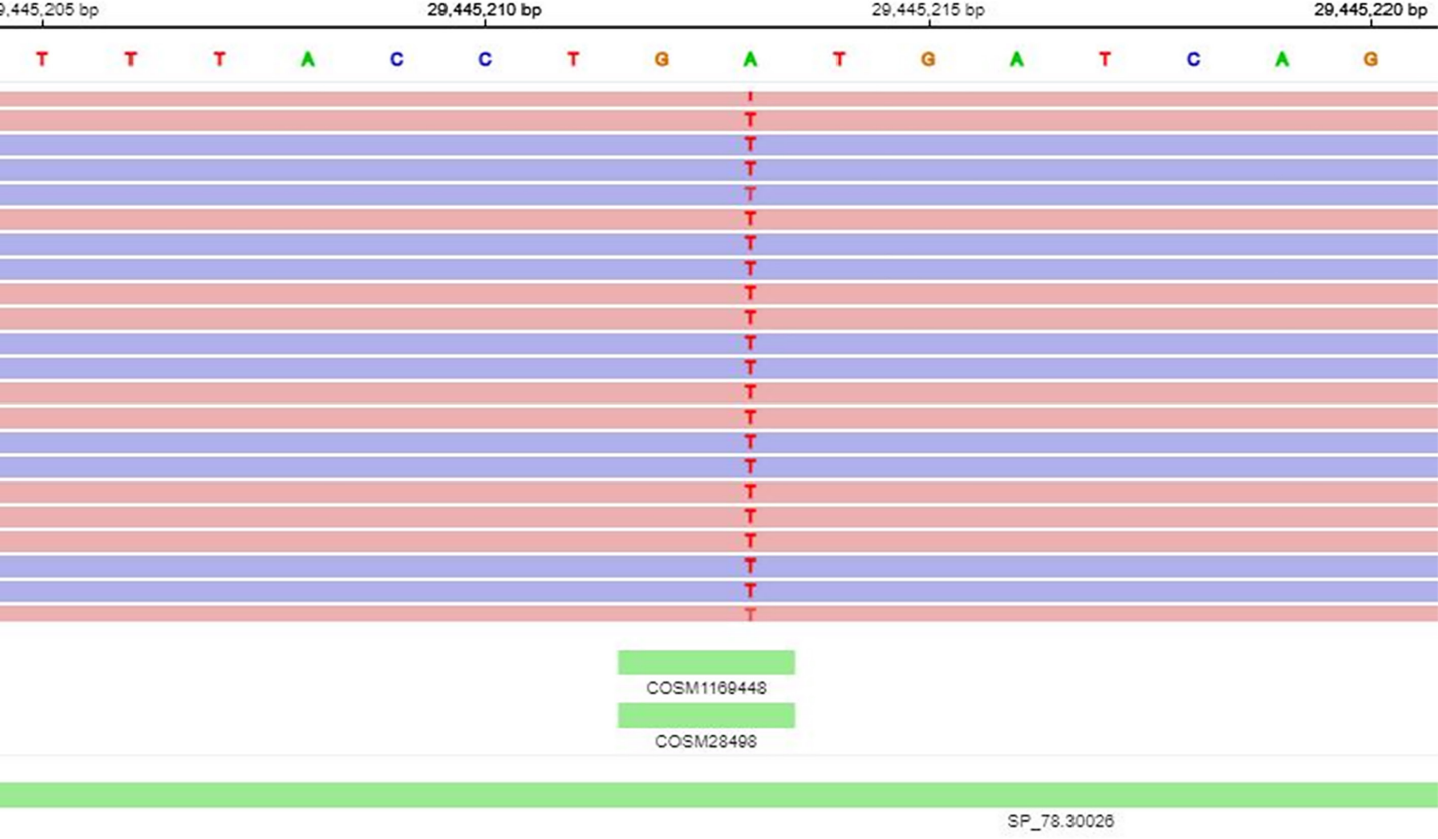
Next generation sequence data from the biopsy following progression on alectinib demonstrating ALK p.I1171N variant.

**FIGURE 5:**
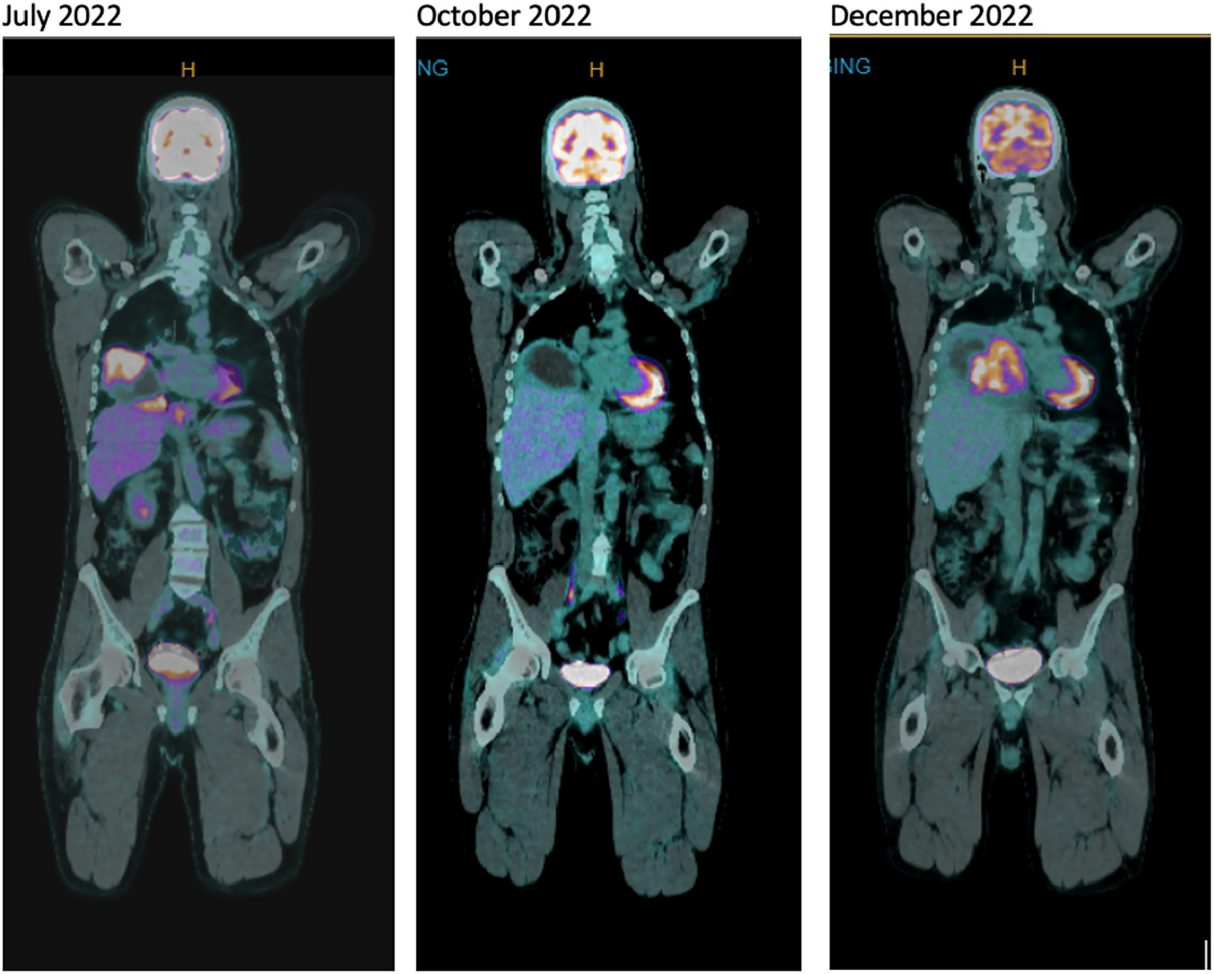
PET scans demonstrating baseline, during response to lorlatinib, and at progression.

Lung biopsy in August 2022 showed persistent *EML4‐ALK* fusion with the emergence of an *ALK* I1171N resistance mutation (Sydpath Oncomine Precision Assay NGS Panel) (Figures 3 and 4). The patient commenced lorlatinib 100 mg daily in August 2022. Again, he had dramatic clinical improvement with resolution of dyspnoea and hypoxia within weeks. PET scan in October 2022 revealed partial response by RECIST criteria with marked reduction in uptake and size of all metastases (Figure 5). Unfortunately, disease progression occurred after 4 months of lorlatinib in December 2022, at which time PET scan showed increased size of lung metastases and a new 120 × 90 mm abdominal soft tissue mass (Figure 5).

**FIGURE 6:**
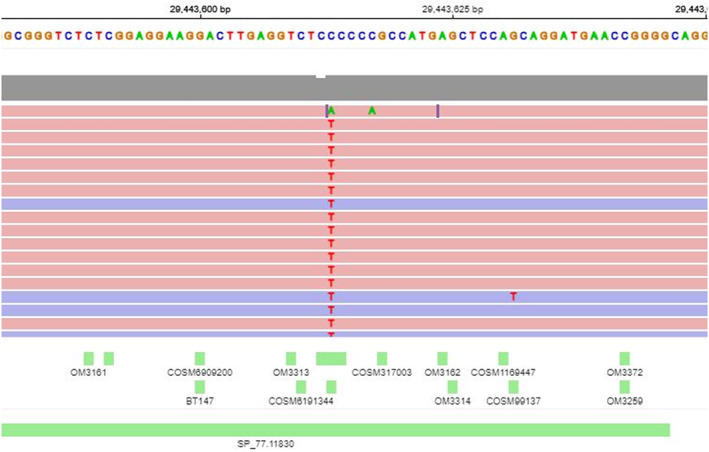
Next generation sequence data from the biopsy following progression on lorlatinib demonstrating ALK gene c.3604G>A p. (G1202R) mutation.

Molecular sequencing of a further lung biopsy in December 2022 showed a new *ALK* kinase domain mutation G1202R (Sydpath Oncomine Precision Assay NGS Panel) (Figure 6). The resistant I1171N mutation disappeared; the *EML4‐ALK* fusion remained.

The patient was managed with palliative radiotherapy (36Gy in 12#) to the abdominal soft tissue mass, while continuing lorlatinib. He was then switched to gemcitabine and docetaxel chemotherapy in February 2023; however, this was ceased after one cycle due to intolerance. The patient was considered for the Phase 1/2 trial of NVL‐655, a fourth generation ALK inhibitor, but unfortunately failed screening due to inability to lie flat for baseline imaging (ClinicalTrials.gov ID NCT05384626). Due to ongoing decline in functional status, the patient did not receive any further systemic therapy. He died of respiratory failure secondary to progressive cancer in April 2023. A summary of treatment is presented in Table 1.

**TABLE 1:** Summary of treatment and development of resistance mutations.

	Treatment	Date commenced	Date progressed	Time on treatment	Resistance mutation identified on progression
First line treatment	Doxorubicin	August 2021	January 2022	4 months	N/A
Second line treatment	Alectinib	February 2022	July 2022	5 months	ALK I1171N mutation
Third line treatment	Lorlatinib	August 2022	December 2022	4 months	ALK G1202R mutation, I1171N no longer present
Fourth line treatment	Gemcitabine + docetaxel	February 2023	February 2023	<1 month	N/A

## 3 Discussion

In this report, we describe a case of metastatic non‐myofibroblastic sarcoma of soft tissue harbouring an *EML4‐ALK* fusion with dramatic clinical and radiologic response to alectinib then lorlatinib and a maintained normal quality of life of 10 months as a result of targeted therapy. To our knowledge, this is the first report describing development of resistance mutations following treatment with ALK TKIs in non‐myofibroblastic sarcoma.

Recent increased understanding of molecular biology has led to refinement in the classification of sarcomas that harbour single driver alterations, however there are few cases of soft tissue sarcoma (STS) with oncogenic driver mutations with effective targeted therapy [3]. *ALK* fusions are rare in metastatic STS, except in myofibroblastic tumours which are enriched for *ALK* rearrangements in up to 50% and have a high response rate to crizotinib [3, 4]. The largest study of *ALK*‐positive mesenchymal tumours (excluding myofibroblastic tumours) is a case series of seven *ALK* IHC positive tumours of which five harboured *ALK* gene rearrangements [5]. Three of these patients received an ALK TKI during their treatment course. One patient with inflammatory epithelioid cell sarcoma who received crizotinib then ceritinib had a partial response to both agents [5]. Two patients (one with undifferentiated sarcoma, the other with smooth muscle tumour of uncertain malignant potential) had stable disease with ceritinib and alectinib respectively. None of these patients underwent repeat biopsy following progression on ALK TKI. There are case reports of ALK positive inflammatory myofibroblastic tumour responding to lorlatinib, but no documented cases of response to lorlatinib in other sarcoma subtypes [6, 7].

There is only limited data to support the use of ALK TKIs in other non‐lung solid organ tumours. In a small phase II study, alectinib has shown activity in melanoma, papillary urothelial carcinoma and colon adenocarcinoma with *ALK* rearrangements, but not in any cases of solid organ tumours with *ALK* mutations or amplification [8]. There have been case reports of response to ALK TKIs in patients with colorectal, myxoid uterine, thyroid, neuroblastoma and pancreas cancers [9, 10, 11, 12, 13, 14, 15]. Biologic rational supports universal sensitivity of ALK TKI's agnostic of organ site of origin, as *ALK* fusions are exquisite tumour drivers.

The duration of response to both alectinib and lorlatinib in the patient was limited to 4–5 months, which is significantly shorter than NSCLC patients treated with first and second generation ALK TKIs, where typical duration of response is 11–36 months [16, 17, 18]. In NSCLC an attenuated benefit to TKI is seen in those harbouring co‐mutations [19, 20]. This is one possible explanation for the short response in our patient who has co‐mutations identified in the initial tumour sample at diagnosis including NTRK1 over‐expression, TERT gain of function mutation and CDKN2A and CDKN2B loss.

In NSCLC, described mechanisms of TKI resistance can be ALK‐dependent (on target), ALK‐independent (off target) or histologic transformation such as small cell or squamous cell [16, 21, 22]. ALK‐dependent resistance refers to resistance mutations within the *ALK* kinase domain and accounts for 30%–40% of resistance mechanisms [16]. The most common resistance mutations in NSCLC patients treated with first and second generation ALK TKIs are G1202R, F1174X, I1171X and G1269A, and the spectrum may differ based on the ALK TKI exposure and selection pressure [23, 24, 25]. ALK‐independent resistance due to activation of bypass tracks (such as RAS, EGFR or ErbB4) has been demonstrated in vitro and in vivo in patients progressing on lorlatinib [26].

This patient developed *ALK* I1171N resistance mutation following treatment with alectinib then a G1202R mutation following lorlatinib. The I1171N resistance mutation has been identified in NSCLC patients as an uncommon resistance mutation following treatment with alectinib [16, 24]. In NSCLC, G1202R is the most common resistance mutation following treatment with the second generation ALK inhibitors (including ceritinib, brigatinib and alectinib), and lorlatinib is effective in overcoming this resistance mutation [24, 27, 28]. It is therefore unexpected that in our sarcoma case, the G1202R mutation developed after lorlatinib treatment and without any co‐mutations identified (I1171N resistance mutation was no longer present).

The mechanism of resistance to lorlatinib in this case is not explained based on the results of the next‐generation sequencing (NGS) panel performed. It would be ideal to expand testing in this situation to a comprehensive genomic profiling panel to evaluate *ALK*‐independent resistance pathways not captured in the NGS lung panel that was used. In addition, an emerging technique is the use of circulating tumour DNA to explore dynamic *ALK* on‐ and off‐target resistance mechanisms not captured in a single site tissue biopsy, noting that in NSCLC, on‐ and off‐target mechanisms often co‐occur [26].

## 4 Conclusion

This case report demonstrates the potential transformative response to targeted therapy in non‐lung solid organ tumours harbouring *ALK* fusions. It highlights the value in performing next generation sequencing in uncommon cancers such as sarcomas as it may identify therapeutic targets of meaningful impact. This is the first description tracking the development of resistance mutations in a patient with non‐myofibroblastic sarcoma therefore adding to what is currently a limited body of evidence in non‐lung ALK‐rearranged cancers.

## Author Contributions

**Isabella Wilson:** conceptualization, methodology, writing – original draft. **Min Qiu:** conceptualization, writing – review and editing, visualization, resources. **Malinda Itchins:** writing – review and editing, resources. **Bin Wang:** visualization, resources. **Min Li Huang:** visualization, resources. **Peter Grimison:** conceptualization, resources, writing – review and editing, supervision.

## Disclosure

Malinda Itchins sits on the advisory board and received honoraria from Roche and Pfizer. Malinda Itchins has received research grants from Pfizer. The other authors have no relevant disclosures.

## Ethics Statement

The investigators obtained informed consent from the patient to publish this case report. There are no identifiable details or photographs included.

## Data Availability

The data that support the findings of this study are available on request from the corresponding author. The data are not publicly available due to privacy or ethical restrictions.
